# Correction to “Core–Shell Gel Nanofiber Scaffolds Constructed by Microfluidic Spinning toward Wound Repair and Tissue Regeneration”

**DOI:** 10.1002/advs.76200

**Published:** 2026-06-17

Yue Dong, Zongkun Ding, Yuting Bai, Ling‐Yu Lu, Ting Dong, Qing Li, Ji‐Dong Liu,* and Su Chen*, “Core–Shell Gel Nanofiber Scaffolds Constructed by Microfluidic Spinning toward Wound Repair and Tissue Regeneration,” *Advanced Science* 11, no. 39 (2024): e2404433, https://doi.org/10.1002/advs.202404433.

The authors regret that the overlap was found in the SEM images in Figures 1c–e. This is an error arising from an oversight during the process of assembling figures; one of SEM images was cropped and magnified with intention to better display the fiber details during data analysis, but eventually mistakenly used during figures compilation for paper preparation. This is a regrettable mistake in figure preparation that does not affect the integrity of the data or reflect an academic misconduct. This correction does not affect any experimental results, discussions, or conclusions reported in the paper. The authors sincerely apologize for any inconvenience caused.

**Corrected Figure 1 and corresponding Figure S2 are shown below**:


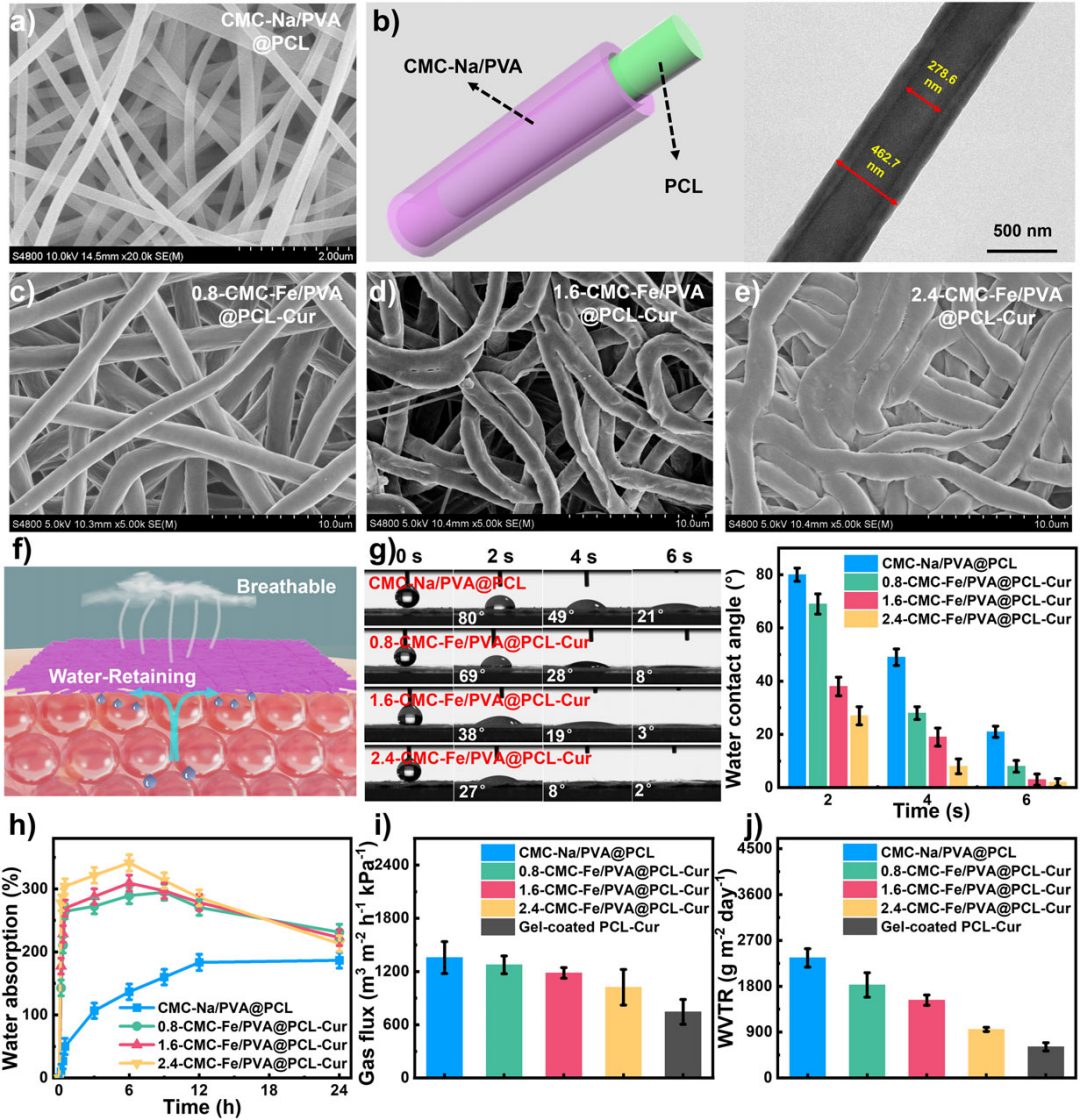


**Figure 1**. a) SEM image of CMC‐Na/PVA@PCL nanofiber. b) Schematic diagram and TEM image of core‐shell 2.4‐CMC‐Na/PVA@PCL nanofiber. c–e) SEM images of gel NFSs. f) Schematic illustration of the surface wettability and breathability of gel NFSs. g) Water contact angle and h) water absorption ratio of CMC‐Na/PVA@PCL nanofiber and gel NFSs with time. i) Nitrogen permeability and j) WVTR of CMC‐Na/PVA@PCL nanofiber, gel NFSs and gel‐coated PCL‐Cur nanofiber.


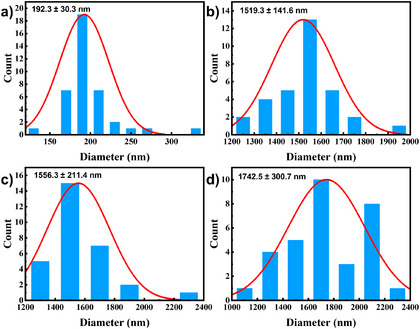


**Figure S2**. Diameter distribution of a) CMC‐Na/PVA@PCL nanofiber, b) 0.8‐CMC‐Fe/PVA@PCL‐Cur gel NFSs, c) 1.6‐CMC‐Fe/PVA@PCL‐Cur gel NFSs and d) 2.4‐CMC‐Fe/PVA@PCL‐Cur gel NFSs.

**Originally published Figure 1 and corresponding Figure S2**:


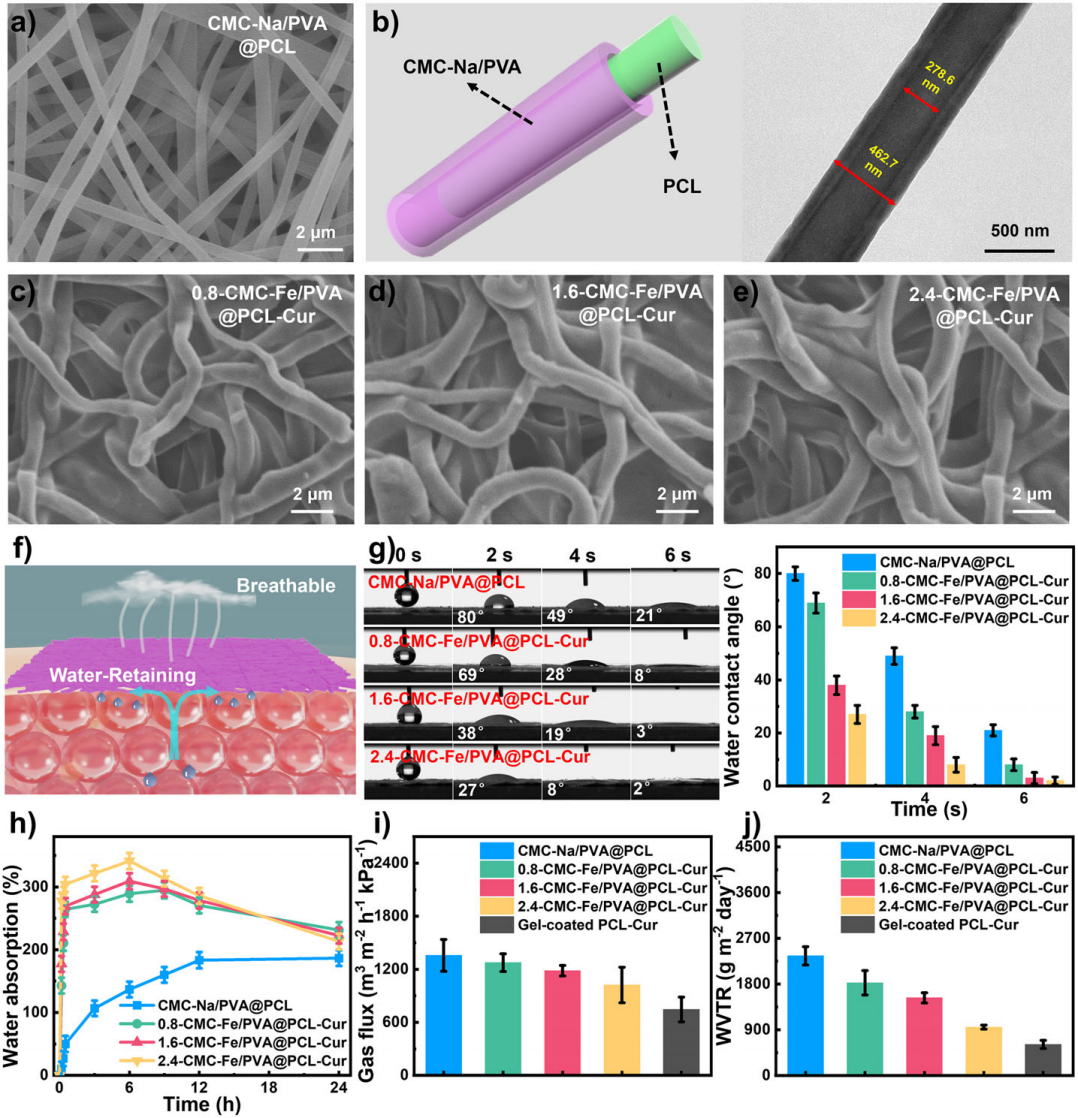


Figure 1


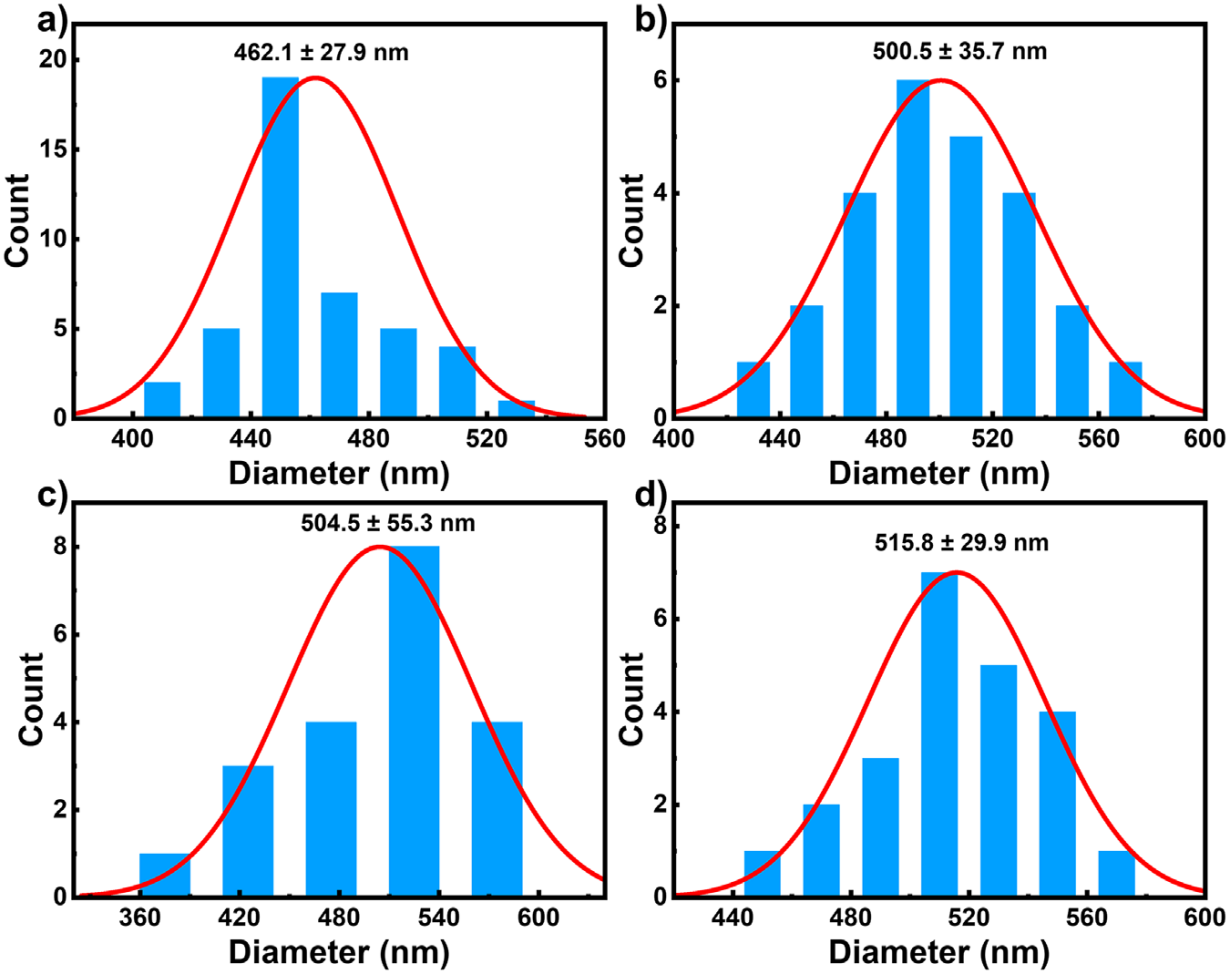


Figure S2

